# Effects of Glycyrrhizin on Multi-Drug Resistant *Pseudomonas aeruginosa*

**DOI:** 10.3390/pathogens9090766

**Published:** 2020-09-18

**Authors:** Nicholas J. Carruthers, Sharon A. McClellan, Mallika Somayajulu, Ahalya Pitchaikannu, Denise Bessert, Xudong Peng, Kylie Huitsing, Paul M. Stemmer, Linda D. Hazlett

**Affiliations:** 1 Institute of Environmental Health Sciences, Wayne State University School of Medicine, 540 E. Canfield Avenue, Detroit, MI 48201, USA; aj7682@wayne.edu (N.J.C.); pmstemmer@wayne.edu (P.M.S.); 2 Department of Ophthalmology, Visual and Anatomical Sciences, Wayne State University, School of Medicine, Detroit, MI 48201, USA; smcclell@med.wayne.edu (S.A.M.); msomayaj@med.wayne.edu (M.S.); gq7089@wayne.edu (A.P.); dbessert@med.wayne.edu (D.B.); kehuits@wayne.edu (K.H.); 3 Department of Ophthalmology, The Affiliated Hospital of Qingdao University, Qingdao 266071, China; doctorpxd@hotmail.com

**Keywords:** *Pseudomonas aeruginosa*, glycyrrhizin, multi-drug resistance, proteomics

## Abstract

The effects of glycyrrhizin (GLY) on multi-drug resistant (MDR) systemic (MDR9) vs. ocular (B1045) *Pseudomonas aeruginosa* clinical isolates were determined. Proteomes of each isolate with/without GLY treatment were profiled using liquid chromatography mass spectrometry (LC-MS/MS). The effect of GLY on adherence of MDR isolates to immortalized human (HCET) and mouse (MCEC) corneal epithelial cells, and biofilm and dispersal was tested. Both isolates were treated with GLY (0.25 minimum inhibitory concentration (MIC), 10 mg/mL for MDR9 and 3.75 mg/mL for B1045) and subjected to proteomic analysis. MDR9 had a greater response to GLY (51% of identified proteins affected vs. <1% in B1045). In MDR9 vs. controls, GLY decreased the abundance of proteins for: antibiotic resistance, biofilm formation, and type III secretion. Further, antibiotic resistance and type III secretion proteins had higher control abundances in MDR9 vs. B1045. GLY (5 and 10 mg/mL) significantly reduced binding of both isolates to MCEC, and B1045 to HCET. MDR9 binding to HCET was only reduced at 10 mg/mL GLY. GLY (5 and 10 mg/mL) enhanced dispersal for both isolates, at early (6.5 h) but not later times (24–72 h). This study provides evidence that GLY has a greater effect on the proteome of MDR9 vs. B1045, yet it was equally effective at disrupting adherence and early biofilm dispersal.

## 1. Introduction

*Pseudomonas aeruginosa (P. aeruginosa)* keratitis is treated by topical antibiotic administration, and in severe cases, by subconjunctival injection. Although antibiotics reduce bacterial burden, tissue damage occurs as a result of a poorly controlled host immune response [1,2]. Additionally, frequent emergence of antibiotic resistant bacteria presents serious challenges for the effective management of keratitis [3] and thus, it is urgent and timely to develop alternative treatment approaches. Resistance to antimicrobials has been observed since the first antibiotics were discovered and many genes that confer drug resistance upon some strains of bacteria pre-date antibiotics by millions of years. However, resistance has increasingly become problematic globally due to overuse of antimicrobials which has increased the rate of resistance development and spread. The lack of new drugs to challenge these new “supermicrobes” exacerbates the problem. Besides health care issues, there is also economic impact of this growing problem, as more than 2.8 million infections caused by antibiotic resistant bacteria occur in the United States yearly [4], costing the US health system USD 20 billion each year in health care costs and costing the US an additional 35 billion dollars a year in lost productivity [5]. *P. aeruginosa*, an opportunistic pathogen, was reported to cause 51,000 healthcare-associated infections/year in 2013 with 13% of these cases resulting from multi-drug resistant (MDR) [6] organisms that are more difficult to treat.

Non-antibiotic-based approaches to prevent and treat bacterial infections are being tested, and could provide alternatives to using antibiotics alone which no longer are effective against MDR *P. aeruginosa* strains. In this regard, glycyrrhizin (GLY) is a glycoconjugated triterpene extracted from the licorice root (*Glycyrrhiza glabra*). It has numerous pharmacological effects [7] and is efficacious in animal models of corneal infection [8,9], sepsis [10], lung [11], and brain [12] injury. It also is used in clinical management of chronic hepatitis [13]. Our purpose is to use proteomics and functional assays to test the effect of GLY on MDR bacteria and whether its effects are similar in a systemic vs. ocular isolate.

## 2. Results

### 2.1. Proteomics

B1045 and MDR9 *P. aeruginosa* isolates were treated with GLY at 0.25 minimum inhibitory concentration (MIC, 10 mg/mL for MDR9 and 3.75 mg/mL for B1045) or vehicle (PBS) with four biological replicates in each group and subjected to bacterial proteomic analysis using orbitrap mass spectrometry with label-free quantification. Four replicate samples of treated and control bacteria for both strains were analyzed (16 samples). The analysis identified 2728 protein groups and quantified 2706. A total of 2264 protein groups had quantitative data for all samples. The median coefficient of variation for protein quantification was 18.9%. GLY treatment did not affect the number of proteins quantified (Supplementary Figure S1A). Median protein abundances had some variability but not so much as to disrupt further analyses (Supplementary Figure S1B).

**Figure 1:**
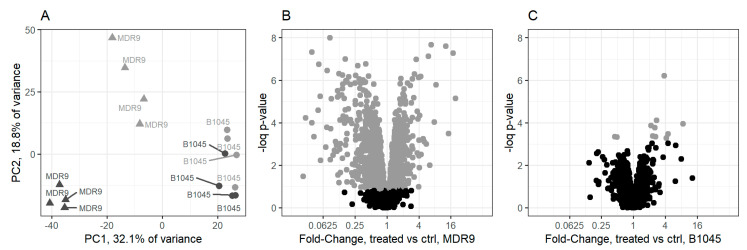
Comparative response of isolates to GLY. MDR9 had a more robust response to glycyrrhizin (GLY) at 0.25 minimum inhibitory concentration (MIC) than B1045. (**A**) PCA showed a greater separation between treatment (dark grey) and control (light grey) in MDR9 (triangles) than in B1045 (circles). A total of 1387 proteins were affected by GLY treatment in MDR9 (*q* < 0.1, moderated *t*-test, n = 4). Affected proteins are represented by light grey points on a volcano plot (**B**) while only 11 proteins were affected in B1045 (**C**). The x-axes of (**B**,**C**) indicate the average fold-change between control and treated samples. The y-axes of (**B**,**C**) indicate -log *p*-values from moderated *t*-test for difference between treatment and control.

Principal component analysis (PCA) was conducted on normalized protein abundances to find linear combinations (components) that explain the most protein variance. The top two components which together account for 50.9% of protein variance separated the isolates indicating that MDR9 and B1045 had substantial differences in protein profiles (Figure 1A). They also demonstrated a difference in how the two isolates responded to GLY treatment. MDR9 was strongly affected by treatment as indicated by the separation of the treated samples from control samples, while B1045 samples were positioned near each other indicating that they did not have a strong response to treatment. Confidence ellipses of 95% for treated and control samples do not overlap for MDR9 samples but do overlap for B1045 samples (not shown).

Statistical analysis of changes in protein abundances between treatment groups confirmed the trends identified in PCA. Overall, 880 proteins were differentially abundant (*q* < 0.1, moderated *t*-test, n = 4) between control MDR9 samples and control B1045 samples indicating substantial differences between the isolates (Table S1). A total of 1387 proteins were affected by GLY treatment in MDR9 samples and just 11 were affected in B1045 (*q* < 0.1, moderated *t*-test, n = 4). Differential responses to GLY between isolates are illustrated in volcano plots (Figure 1B,C). The distribution of *p*-values for treatment-induced differences indicates that 783 proteins were affected by treatment in B1045 but most could not be identified at a reasonable false discovery rate (Supplementary Figure S2A,B). The PCA and *t*-test results were in agreement that there were substantial differences in the proteome of the two isolates and that they had different responses to GLY treatment. MDR9 had a large magnitude response while B1045 had a small response.

Systems that responded to GLY treatment or that were different between B1045 and MDR9 control samples were investigated using protein set analysis. First, manually curated protein sets were compiled and tested for enrichment in affected proteins using PIANO [14]. A set of 55 manually selected proteins were assigned to three gene sets: antibiotic resistance, biofilms/adherence and virulence/quorum. Twenty-six of those proteins were identified in our dataset. T-statistics for treatment-induced differences within each isolate as well as between isolates along with the three gene sets were submitted to PIANO software for analysis. The antibiotic resistance set, which consisted of two proteins, was decreased in GLY-treated samples relative to control MDR9 and also less abundant in B1045 controls relative to MDR9 controls (Table 1).

**Figure 2:**
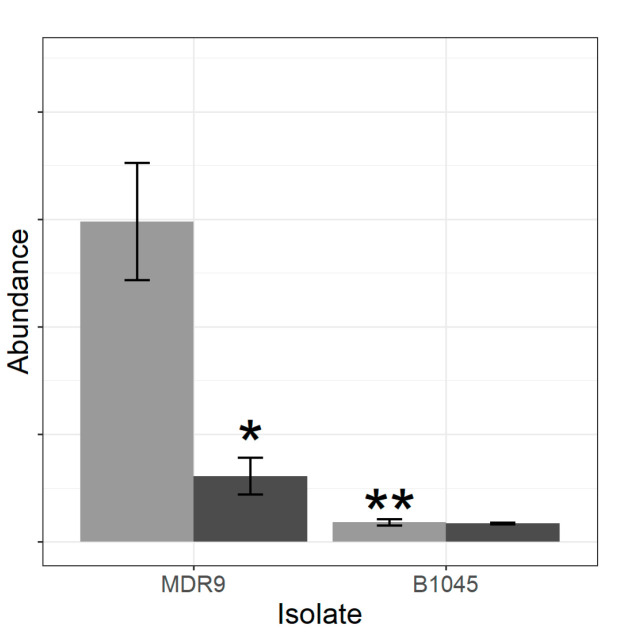
Abundance of beta-lactamase by isolate and treatment was identified from selected proteins of interest as being decreased in GLY-treated MDR9 and control B1045 relative to control MDR9. Light grey indicates control and dark grey shows GLY-treated samples. Bars indicate mean + standard deviation, n = 4. * Indicates differences after GLY treatment where only MDR9 was significant. ** Indicates differences between control isolates (moderated *t*-test, *q* < 0.1, n = 4).

That effect was completely due to the differences between groups in beta-lactamase protein abundance (Figure 2).

**Figure 3:**
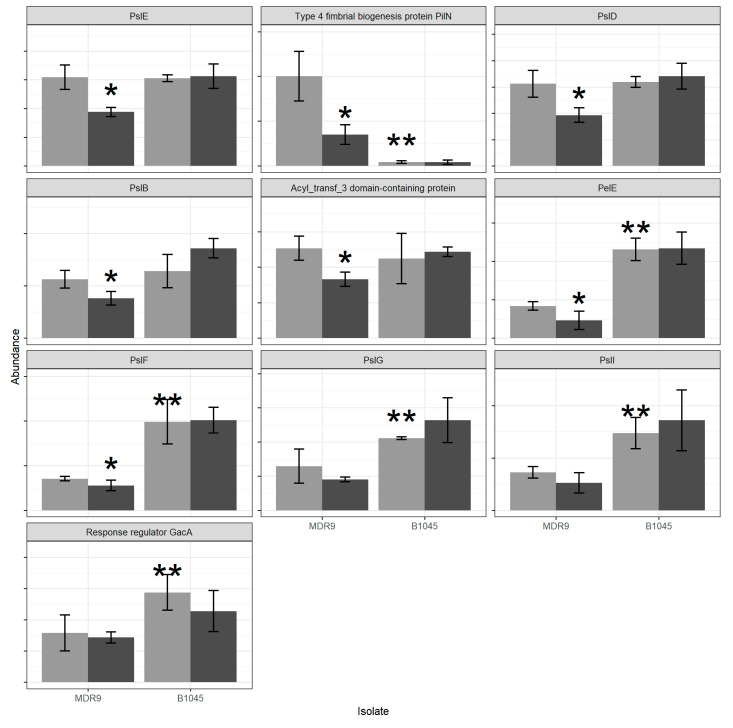
Proteins relating to biofilm/adherence. Light grey indicates control samples and dark grey indicates GLY-treated samples. Bars indicate mean + standard deviation, n = 4. Graphs are sorted by *p*-value for a GLY treatment effect in MDR9 with the lowest value at the top left. * Indicates differences from control after GLY treatment and ** shows differences between the two control isolates (moderated *t*-test, *q* < 0.1, n = 4; none of the proteins were affected by GLY treatment in B1045).

Figure 3 shows proteins (PslE, PilN, PslD, PslB, Acyl domain containing protein, PelE, PslF, PslG, PslI, and GacA) in the biofilm/adherence category which, as a set, were decreased in abundance in GLY-treated MDR9 samples relative to controls and were significant for all proteins (PslE, PilN, PslD, PslB, Acyl domain containing protein, PelE and PslF) except for PslG, PslI and Gac. These proteins were not affected by GLY treatment in B1045. Significant differences between untreated isolates also were found for: PilN (reduced), while the others PelE, PslF, PslG, PslI and GacA were increased in B1045 vs. MDR9.

In addition, the String database functional enrichment tool [15] was used to identify unanticipated systems that were affected by GLY treatment or that were different between untreated isolates. T-statistics for treatment-induced differences in each isolate as well as between isolates were also submitted to the functional enrichment tool in the String database. Overall, 27 protein sets were differentially abundant between control samples of either isolate, 53 sets were affected by GLY treatment in MDR9 samples and 7 were affected by that treatment in B1045 samples (adjusted *p* < 0.01, Supplementary Table S2). The String-identified protein sets that were affected by GLY treatment could be separated into five clusters based on common protein membership. Representative sets for each cluster are listed in Table 2.

**Figure 4:**
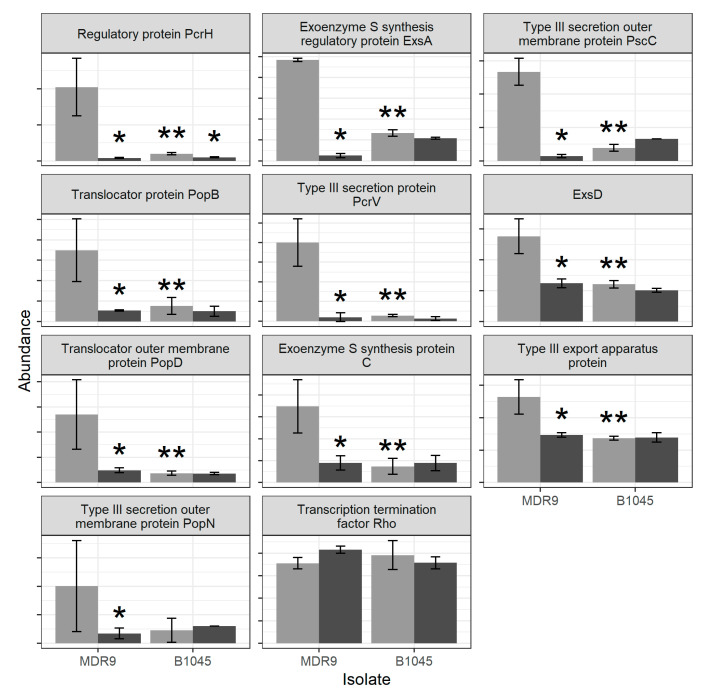
Proteins described in Galle et al. [16] or in String cluster CL:5839 (“mixed, include bacterial secretion system, and Tir chaperone protein (CesT) family”) from the *P. aeruginosa* string network. Light grey indicates control samples and dark grey indicates GLY-treated samples. Bars indicate mean +/− standard deviation, n = 4. Graphs are sorted by *p*-value for a GLY treatment effect in MDR9 with the lowest value at the top left. * Indicates differences between MDR9 or B1045 controls after GLY. ** Indicates differences between the two control isolates. (moderated *t*-test, *q* < 0.1, n = 4).

Abundances for proteins involved in bacterial secretion and the Tir chaperone protein (CesT) family are shown in Figure 4 and include: PcrH, ExsA, PscC, PopB, PcrV, ExsD, PopD, synthesis protein C, type III export apparatus, PopN and termination factor Rho. All of them were significantly reduced (*) in GLY-treated MDR9 compared with control except transcription termination factor Rho. GLY treatment of B1045 only decreased PcrH significantly (*) compared with control. Differences between isolates also are indicated (**).

### 2.2. Bacterial Adherence to Mouse Corneal Epithelial Cells (MCEC) and Human Corneal Epithelial Cells (HCET)

**Figure 5:**
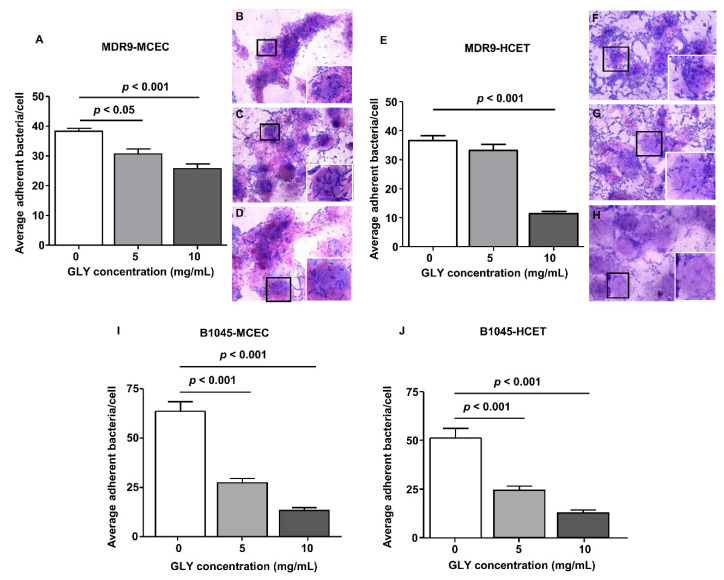
MDR9 and B1045 adherence to mouse corneal epithelial cells (MCEC) (**A**–**D**,**I**) and human corneal epithelial cells (HCET) (**E**–**H**,**J**). The average number of MDR9 bacteria per cell was decreased for MCEC at 5 and/or 10 mg/mL GLY (**A**,**E**), but binding to HCET was reduced only at 10 mg/mL. Adherent bacteria are shown in images taken using a bright field microscope with no GLY (**B**,**F**) and using GLY (5 mg/mL (**C**,**G**) and 10 mg/mL (**D**,**H**)) treatment. An inset is shown in (**B**–**D**,**E**–**H**), and represents an enlargement of the cells in each photomicrograph that are outlined by a black box. Adherence of B1045 to MCEC and HCET was significantly reduced when the isolate was treated with either 5 or 10 mg/mL GLY. All data are mean ± SEM and were analyzed using a Bonferroni’s multiple comparison test (n = 100 cells/group). Magnification = ×20 µm, inset magnification = ×40 µm.

Immortalized human corneal epithelial (HCET) and primary corneal epithelial cells from C57BL/6 mice were used to test the ability of MDR9 and B1045 to adhere to these cells as adherence is the first step in biofilm formation. The data are shown in Figure 5. Bacteria were untreated (0 GLY) (Figure 5B,F) or treated with 5 (Figure 5C,G) or 10 mg/mL GLY (Figure 5D,H) just before application to MCEC (Figure 5B,C) or HCET (Figure 5G,H), and incubated for 3 h.

Wright–Giemsa staining revealed bacteria adherent to MCEC and HCET in both control (Figure 5B,F) and GLY-treated groups (Figure 5C,D,G,H). The non-ocular isolate, MDR9 combined with GLY immediately before application to the murine cultured cells had significantly decreased numbers of adherent bacteria at concentrations of both 5 (*p* < 0.05) and 10 mg/mL (*p* < 0.001) (Figure 5A). MDR9 adherence to HCET was significantly reduced compared to control (no GLY) adherence after 10 mg/mL GLY treatment only (*p* < 0.001, Figure 5E). Adherence to MCEC and HCET of the ocular isolate, B1045, was significantly reduced by both 5 and 10 mg/mL GLY (*p* < 0.001 for all, Figure 5I,J).

### 2.3. Biofilm and Dispersal with GLY

**Figure 6:**
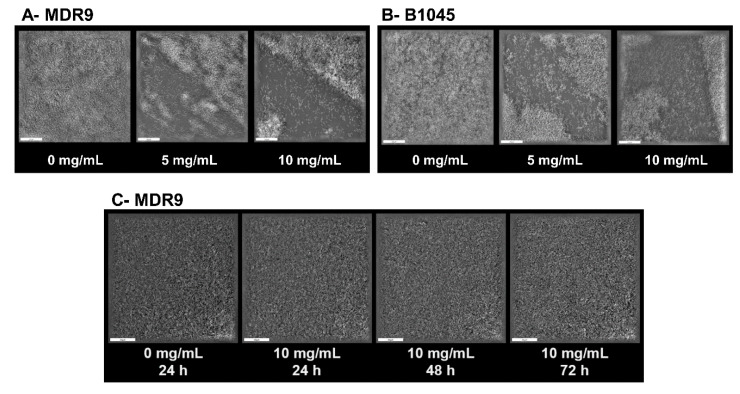
Tomographic images show dispersal of a biofilm formed by MDR9 (**A**) and B1045 (**B**) at 6.5 h after treatment with GLY at 0 (left panel), 5 (middle panel) or 10 mg/mL (right panel), respectively. Biofilm dispersal of B1045 after either 5 (middle panel) or 10 (right panel) mg/mL GLY treatment vs. control (0 GLY, left panel) was similar. MDR9 (**C**) images are shown after GLY treatment with 10 mg/mL at 24, 48 and 72 h (more mature biofilms) compared to a 24 h control (all controls appeared the same). GLY did not disperse the more mature biofilms.

To interrogate the effects of GLY on a formed biofilm in culture at an early (6.5 h) (Figure 6A,B) and later 24–72 h (Figure 6C) biofilm, 3D tomographic microscopy was used. One hour after treatment of MDR9 (Figure 6A) with GLY (5 mg/mL, middle panel), the biofilm began to disperse and at 10 mg/mL (right panel), dispersal increased to about 50% vs. control (no GLY, left panel). Figure 6B shows 3D tomographic microscopy images of a similarly established and treated B1045 biofilm. Since early dispersal was similar between the two isolates, we similarly tested MDR9 only and at 10 mg/mL concentration on a more mature biofilm (24–72 h) (Figure 6C). GLY did not disperse any of the later formed biofilms.

## 3. Discussion

*Pseudomonas aeruginosa* is an opportunistic pathogen that is one of the most frequent causes of bacterial keratitis worldwide [17]. Due to the alarming rate at which *P. aeruginosa* antibiotic resistance is emerging, the need for new and effective antimicrobials is crucial. Glycyrrhizin (GLY), a glycoconjugated triterpene isolated from the root of *Glycyrrhiza glabra*, is a naturally occurring anti-inflammatory/antimicrobial that has been shown to be effective in many animal models of disease, including keratitis [8,9], colitis [18], sepsis [10], lung injury [11] and clinically in cases of hepatitis [13].

GLY’s efficacy against *P. aeruginosa* keratitis has been examined recently utilizing several clinical isolates; KEI1025, R59733, 070490, G81007, and RS1. GLY had a minimum inhibitory concentration (MIC) of 40 mg/mL for each of the clinical isolates as well as two lab strains (PAO1 and 19660) tested [8,19,20]. The MIC of multi-drug resistant strain MDR9, isolated from sputum also was 40 mg/mL, but MIC was reduced to 15 mg/mL for the ocular isolate B1045 [21], suggesting that GLY may not affect all MDR similarly.

One of the highest and most significant differences induced by GLY treatment was the reduction of beta-lactamase only in the MDR9 isolate. Increased beta-lactamase, an antibiotic inactivating enzyme, is employed by *P. aeruginosa* along with outer membrane permeability and increased expression of efflux pumps to induce resistance [22] and so its reduction by GLY would lead to decreased resistance. In addition, when comparing untreated isolates, B1045 had significantly less beta-lactamase than MDR9. Furthermore, a study in Japan determined that of a possible 800 beta-latamases, 120 different ones were identified in *P. aeruginosa* clinical isolates [23], suggesting that GLY may have specificity of target or amount. Some of these expressed extended-spectrum lactamases (ESBLs) that are able to provide a high degree of resistance to the majority of beta-lactams (penicillins, cephalosporins and aztreonam) [24,25]. Beta-lactamase-associated resistance is also subject to the efficiency of antibiotic penetration and accumulation that is regulated by other intrinsic resistance factors. It is not unreasonable then to theorize that the beta-lactamase profiles will differ between isolates [26] explaining the disparate levels of beta-lactamase between MDR9 and B1045. Past studies from our laboratory, including a proteomic comparison of RS1 (a non-ocular, drug resistant clinical isolate) and PAO1 showed that the gene *ampC*, which is responsible for beta lactamase production, was detectable in RS1 but not PAO1 [20]. Additional studies showed GLY treatment had an effect on other intrinsic resistance mechanisms, namely increase in membrane permeability in MDR9 and significant reduction in expression of major resistance-nodulation-division (RND) efflux pump genes in both MDR9 and B1045 [21]. Combined with the finding that GLY reduces beta-lactamase in MDR9, but not B1045, the premise that GLY can alter multi-drug resistant mechanisms differentially between isolates appears supportable.

Production of antibiotic inactivating enzymes is not the only method *P. aeruginosa* employs to achieve antibiotic resistance. Adaptive resistance, involving adherence to living or non-living surfaces and formation of an aggregate of bacteria embedded in a matrix of secreted polysaccharides, proteins, and extracellular DNA known as biofilm, obstructs and limits antibiotic access to the bacteria [22]. In this regard, MDR9 vs. B1045 differed again in that GLY significantly decreased proteins associated with biofilm formation and adherence only in MDR9. In addition, comparing the two isolates (not treated with GLY) significant differences in biofilm components were seen, indicating that all MDR isolates are not similar.

Biofilm formation occurs in five distinct phases: (i) reversible attachment (>0 min), (ii) irreversible attachment (2 h), (iii) maturation-1 (3 days), (iv) maturation-2 (6 days), and (v) dispersion (9–12 days). Bacteria express multiple phenotypes during these stages with the average change in proteins between stages being 35% (~525 proteins) [27]. Biofilm is composed of a heterogeneous, metabolically active population of bacteria compared to a homogeneous population in planktonic bacteria. This is of importance, since it has been shown that there is only a 1% (~73 of 5500) difference in genes when comparing planktonic and biofilm cells. In the biofilm, of that 1% difference, 0.5% of the genes are activated, and 0.5% are repressed. For example, in a biofilm, genes for synthesis of flagella and pili are repressed and efflux pump genes are not activated [28]. With regard to pili, GLY reduced protein PilN in MDR9 and untreated B1045 had significantly less PilN than untreated MDR9 both of which could decrease initiation of a biofilm by affecting bacterial attachment. For attachment, most environmental and non-cystic fibrosis (CF) clinical isolates secrete Psl or Pel, polysaccharide components of the biofilm [29]. In this regard, GLY reduced PslB which could also affect attachment. Support for this proposal is provided by a study using a PAO1 *psl* mutant which was found completely deficient in attachment [30]. Another study in PAO1 specifically disrupted *pslA* and *pslB,* which also severely compromised biofilm initiation, and the study carefully ruled out motility defects or biosynthesis of endotoxin (LPS) [31].

Others have shown that mutations in various *psl* genes also resulted in clones impaired in their ability to form surface-attached communities [32]. In fact, overproduction of Psl results in hyper-biofilm structure and architecture that is similar to that seen in *P. aeruginosa* variants with elevated *psl* and *pel* [33]. In our study, GLY reduced PelE only in MDR9 and was the only one of seven Pel proteins detected in either isolate. PelE, an inner membrane protein, may function as a scaffold protein similar to AlgK and help in assembly of a secretion complex through interaction with PelA and PelB [29]. Finally, another biofilm protein, GacA was significantly higher in B1045 vs. MDR9, however GLY treatment had no significant effect on either isolate suggesting that this biofilm protein is not a target of GLY. This is unfortunate, however, because when *gacA* activity was disrupted by mutation in strain PA14, biofilm formation was reduced 10-fold compared to wildtype strain. In that study, neither flagellar- or pili-mediated attachment appeared to be affected by *gacA* mutation and PA14 remained highly resistant to antibiotics [34]. In contrast, B1045 had higher amounts of PslF, PslG, PslI, and PelE than MDR9 which suggests that it might have more adherence capacity, however, when tested by an adherence assay (to corneal epithelial cells) GLY was equally effective in decreasing adherence in both isolates. Alternatively, GLY which decreased PilN in MDR9 may have reduced type IV pili which account for about 90% of *P. aeruginosa* adherence ability [35]. The diminished levels of PilN present in B1045 (Figure 3) in the absence of GLY treatment could produce the same effect.

String analysis was used to pinpoint unanticipated systems associated with virulence that were also significantly affected by GLY treatment or had significantly disparate expression between the two isolates (type III secretion (T3SS, Table 2). This system allows transfer of effector toxins directly into the host cell [16]. It is highly regulated and therefore, changes in the components of its machinery that reduce functionality also reduce infectivity associated with severe disease [36,37]. GLY significantly reduced proteins associated with type III secretion, including PcrH (both isolates) and PopB, PopD, PcrV, PopN, and PscC proteins only in MDR9. Others have shown that absence of PcrH resulted in a deficiency in *P. aeruginosa* translocation of PopB and PopD, but despite this deficiency, there was no regulatory defect in type III secretion function [38]. This could be due to the fact that the *P. aeruginosa* genome possesses the ability to encode a high number of regulatory factors and the need for chaperone-dependent regulation by PcrH may be overcome. GLY reduction of PopB, PopD, and PcrV in MDR9 should lead to decreased virulence as these proteins interact with one another to form a translocation pore, making them essential for type III secretion function [39]. PcrV serves as a platform for the translocation pore and is found in both the bacterial cytoplasm and localized extracellularly which could allow its participation in more than one component of *P. aeruginosa* infectivity [40]. Others have shown that neutralization of the extracellular form of PcrV by antibodies blocked toxin injection and enabled phagocytosis of *P. aeruginosa* by macrophage cell lines [41]. Another study by Yang et al. [42] using mutant strains lacking *pcrV* or *popN* indicated that both of them function as inhibitors of the type III secretion apparatus. GLY inhibition of PscC, an outer membrane protein that (along with PscW) forms a channel and provides a path for secreted proteins to access the cell exterior/surface would also reduce virulence. Transcription of type III secretion system genes is regulated by the transcriptional regulator ExsA which is dependent upon coupling with one of three additional proteins ExsC, ExsD, or ExsE [16]. GLY treatment significantly reduced ExsA and ExsD only in MDR9; their constitutive expression was lower in B1045 vs. MDR9. These are significant as even minor changes in the levels of a component of the regulatory cascade (ExsA, ExsC, ExsD, or ExsE), as little as three-fold, can have extreme effects on type III secretion system gene expression [43]. ExsA autoregulates its own expression [44] but can be disrupted by the steric hindrance of ExsD, an anti-activator that inhibits the self-association and DNA-binding activity of ExsA [45]. Transcription termination factor Rho was the only protein that was significantly increased by GLY treatment in MDR9. This event could lead to increased operon transcription suppression which others have shown in studies using microarray, ChIP-Seq, and proteomics assays [46].

No bacterial target protein for GLY action or detailed mechanism of action can be identified from these data. GLY-induced changes in protein abundance were approximately equally split between increased and decreased protein abundance (Figure 1B,C) suggesting that GLY does not act as a universal inhibitor of transcription or translation. Further studies will be needed to identify the molecular targets of GLY.

In summary, proteomics has identified major differences between the systemic and ocular isolates after GLY treatment and between the two non-treated control isolates as well. Despite the many differences identified in proteomic analysis, when GLY treatment was delayed post biofilm formation, it effectively caused dispersal in a similar manner in both isolates early (6.5 h), but not in more mature (24–72 h) biofilms.

## 4. Materials and Methods

### 4.1. Bacterial Culture

*P. aeruginosa* strains MDR9 (isolated from sputum; Detroit Medical Center, Detroit, MI, USA) and B1045 (clinical keratitis isolate provided by Regis Kowalski, PhD, Charles T. Campbell Ophthalmic Microbiology Laboratory, School of Medicine, University of Pittsburgh, Pittsburgh, PA, USA) were grown in peptone tryptic soy broth (PTSB) medium at 37 ℃ in a rotary shaker water bath at 150 rpm for 18 h to an optical density (measured at 540 nm) between 1.3 and 1.8. Bacteria were pelleted by centrifugation at 5500 g for 10 min, washed once with sterile saline, recentrifuged, resuspended, and diluted in sterile saline (0.85%, pH = 7.4).

### 4.2. GLY Treatment For Proteomics

GLY solution (5 mL/tube, 4 tubes total) was prepared in PTSB at a concentration equal to ¼ the MIC (10 mg/mL for MDR9 and 3.75 mg/mL for B1045) [8] and 10 µL of each bacterial culture (adjusted to 1.5 × 10^8^ cfu/mL using the 0.5 McFarland standard) was added to each tube and incubated at 37 ℃ for 18 h. Bacteria were pelleted and washed one time in sterile saline. Control samples were grown in PTSB with no GLY.

### 4.3. Proteomics

In total 16 bacterial pellets (MDR9 vs. B1045) in 2 sets of 4 biological replicates per group were submitted for proteomic analysis. Pellets were solubilized in 100 ul of 40 mM triethylammonium bicarbonate (TEAB, Sigma-Aldrich, St. Louis, MO, USA), 2% lithium dodecyl sulfate (LiDS, Sigma-Aldrich, St. Louis, MO, USA) and heated at 95 °C for 5 min. Non-soluble particulates were filtered away with Handee Spin Columns (Pierce Thermo Fisher Scientific, Rockford, IL, USA) and protein amounts were determined by the BCA Protein Assay method (Pierce). Samples were then reduced with 5 mM dithiothreitol (DTT), and alkylated with 15 mM iodoacetamide (IAA) under standard conditions. Excess IAA was quenched with an additional 5 mM DTT. An amount of 30 µg of each sample was aliquoted, acidified with 1.2% phosphoric acid, followed by a 7-volume addition of 90% methanol (MeOH), 100 mM TEAB binding buffer and loaded onto S-Trap Micro Columns (Protifi, Farmingdale, NY, USA). Columns were washed 2 times with 90% MeOH, 100 mM TEAB buffer with spins at 4000 g for 2 min each. Samples were digested on-column using sequencing-grade trypsin (Promega, Madison, WI, USA) in 40 mM TEAB, 2 mM borax at for 1 h, then transferred to incubate at 37 ℃ for continued digestion overnight. The next day, peptides were eluted off the columns using 50 mM TEAB.

The peptides were separated by reversed-phase chromatography (Acclaim PepMap100 C18 column, Thermo Scientific, Rockford, IL, USA), followed by ionization with the Nanospray Flex Ion Source (Thermo Scientific), and introduced into a Q Exactive mass spectrometer (Thermo Scientific) using 1 h chromatography gradients. MS1 scans were collected at 70K resolution and MS2 scans at 17.5K. Ten data-dependent MS2 scans were collected after each MS1 scan using higher energy collisional dissociation.

Raw files were searched using Thermo Proteome Discoverer version 2.4.0.305 (Thermo Scientific). Peptide sequences were from the Uniprot Pseudomonas aeruginosa proteome UP000002438 (downloaded 2019-11-21, 7333 sequences) requiring fully tryptic peptides at least 6 residues long with, at most, 2 missed cleavage sites. Precursor mass tolerance was 20 ppm and fragment tolerance was 0.02 Da. Methionine oxidation, glutamine and asparagine deamidation and protein *N*-terminal acetylation were variable modifications and cysteine carbamidomethylation was a fixed modification. Peptide identifications were accepted at a 1% false discovery rate as determined by a reversed database. Protein quantitative values were calculated as the sum of precursor areas under the curve.

### 4.4. Bacterial Adherence Assay

MDR9 and B1045 were prepared as described above and bacterial growth examined as described before [8]. Briefly, bacterial cultures were grown overnight at 37 ℃ in PTSB for 18 h. Bacterial suspensions were prepared in sterile saline, adjusted to a concentration of 1.5 × 10^8^ cfu/mL using the 0.5 McFarland standard. Transfected human corneal epithelial cells (HCET, cell line 10.014 pRSV-T, gift of Dr. Gabriel Sosne) cultured in KBM (Lonza, Walkersville, MD, USA) with growth factors and primary B6 mouse corneal epithelial (MCEC) cells were grown as described before [47]. Each cell line, 2 × 10^5^ cells/mL of complete media [47], was seeded onto Falcon polystyrene tissue culture-treated glass chamber slides (4 chambers/slide) and incubated overnight. Cell chambers were washed twice with D-PBS, then 1 mL fresh media was added without antibiotics. Bacteria (washed and reconstituted in sterile saline) were combined with GLY (0, 5 and 10 mg/mL) immediately before application to the chambered slides in a volume equal to 10 multiplicity of infection (MOI) or 2 × 10^6^ bacteria/cell. Each slide was incubated for 3 h at 37 ℃ under aerobic conditions. Then, slides were washed three times with sterile PBS. Air dried slides were stained (Wright–Giemsa, Sigma-Aldrich, St. Louis, MO, USA) for 30 s followed by PBS (0.5 mL for 1 min). Then, chambers were gently decanted and washed with PBS. After air drying, chambered slides were mounted with permount, observed (brightfield microscope) and photographed as before [19]. Bacteria adhered to cells were quantified (n = 100 cells/group), averaged and reported as number of adherent bacteria per cell [19].

### 4.5. Biofilm Dispersal by Glycyrrhizin

The effects of GLY on dispersal of a biofilm were visualized using a modification of previously described methods [48,49]. Briefly, MDR9 and B1045 were grown as described above. Dishes (35 mm) with a 23 mm glass surface area (World Precision Instruments, Sarasota, FL) were inoculated with bacteria at a concentration of 1 × 10^6^ in 1 mL PTSB, and biofilm grown at 37 ℃ with shaking at 50 rpm in a MaxQ 4000 orbital shaker (Thermo Fisher Scientific, Waltham, MA, USA) for 6.5 (both isolates) and only MDR9 at 24, 48 and 72 h. Media containing planktonic bacteria was removed, and fresh media (0 mg/mL), or media containing 5 or 10 mg/mL GLY was added to the dishes and incubated for 1 h at 37 ℃ with shaking at 100 rpm for MDR9 and 50 rpm for B1045. Media was removed and the dishes washed 2 times with D-PBS. Biofilm dispersal was shown with 3D tomographic images captured using a NanoLive 3D Tomographic microscope (Nexus LLC, Washington, DC, USA).

### 4.6. Statistical Analysis

For all mass spectrometry data, statistical analysis was carried out in R version 3.6.2. Channels were normalized to all have the same median abundance and log2 transformed. Differences between treatment groups were identified using a moderated *t*-test [50]. Proteins that were present in just one treatment group but not the other could not be analyzed using a *t*-test. Instead they were tested for between-group differences using Poisson regression of spectral counts. *p*-values from the two tests were pooled and converted to q-values [51]. T-statistics from the *t*-test and z-scores from the regression were also pooled and then submitted for protein set analysis. Protein set analysis was conducted using PIANO [14] for manually curated sets and the String database functional enrichment tool [15]. The entire analysis was conducted in duplicate. The results of one analysis are shown here and all reported findings were reproduced in the second (not shown). For comparison of three or more groups, a 1-way ANOVA followed by the Bonferroni’s multiple comparison test (GraphPad Prism) was used for analysis. For each test, *p* < 0.05 was considered significant and data were shown as mean ± SEM. All experiments were repeated at least once to ensure reproducibility.

## Figures and Tables

**Table 1:** Mean *t*-statistics for proteins in manually curated protein sets.

Set Name	Proteins in Set	GLY vs. ctrl MDR9	GLY vs. ctrl B1045	MDR9 vs. B1045
Antibiotic Resistance	2	* −5.12	0.38	* 11.98
Biofilms/Adherence	17	* −1.77	0.32	−0.63
Virulence/Quorum	7	−0.77	0.99	−0.31

GLY = glycyrrhizin, MDR9 = Multi-Drug Resistant 9, FDR = False Discovery Rate. * Indicates significant, FDR adjusted *p* < 0.1.

**Table 2:** Selected protein sets from the String database functional enrichment tool analysis of GLY treated vs. control MDR9.

Set ID	Description	Score *	FDR	Proteins in Set	Similar Pathways
CL:7881	mixed, incl.Biosynthesis of siderophore group				
	nonribosomal peptides, and Acetyl-coenzyme A	−5.86	3.07 × 10^−6^	8	15
	transporter 1				
CL:5839	mixed, incl Bacterial secretion system,				
	and Tir chaperone protein (CesT) family	−3.32	4.61 × 10^−6^	13	14
CL:126	Ribosome	1.28	6.91 × 10^−6^	48	17
CL:1605	Citrate cycle (TCA cycle)	2.19	6.40 × 10^−4^	16	4
CL:2538	Quinone, and Thioredoxin-like [2Fe-2S]				
	ferredoxin	2.62	8.10 × 10^−4^	11	3

* Positive score indicates increased abundance in GLY-treated samples and negative indicates decreased abundance.

## Supplementary Materials

The following are available online at https://www.mdpi.com/2076-0817/9/9/766/s1, Figure S1: Number of proteins quantified and median abundance for each sample, Figure S2: *p*-value histograms, Table S1: Quantitative values and statistics for all proteins quantified in this study, Table S2: Protein sets identified by the String database functional enrichment tool.
