# Supplementary material for: Effects of Glycyrrhizin on Multi-Drug Resistant Pseudomonas aeruginosa
Source: Pathogens. 2020 Sep 18;9(9):766. doi: 10.3390/pathogens9090766 (PMC7557769; doi:10.3390/pathogens9090766)
Supplement: Supplementary file 1 [file pathogens-09-00766-s001.zip › Supplemental Figure S1.docx]

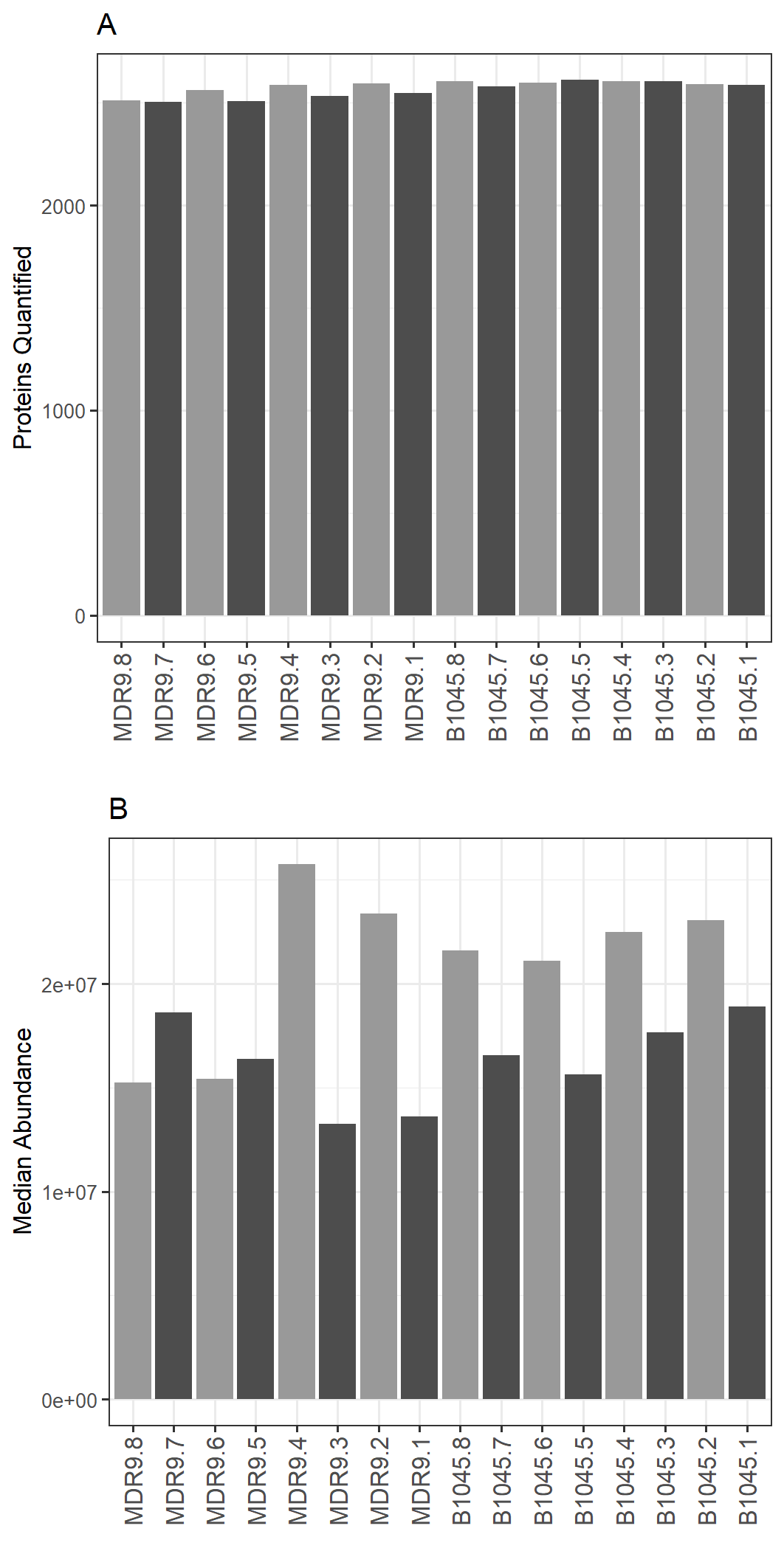


Supplemental Figure S1. Number of proteins quantified and their median intensity are not different between isolates or affected by GLY treatment. Light grey bars are control samples, dark grey bars are treated.
