# Supplementary material for: Effects of Glycyrrhizin on Multi-Drug Resistant Pseudomonas aeruginosa
Source: Pathogens. 2020 Sep 18;9(9):766. doi: 10.3390/pathogens9090766 (PMC7557769; doi:10.3390/pathogens9090766)
Supplement: Supplementary file 1 [file pathogens-09-00766-s001.zip › Supplementary Figure S2.docx]

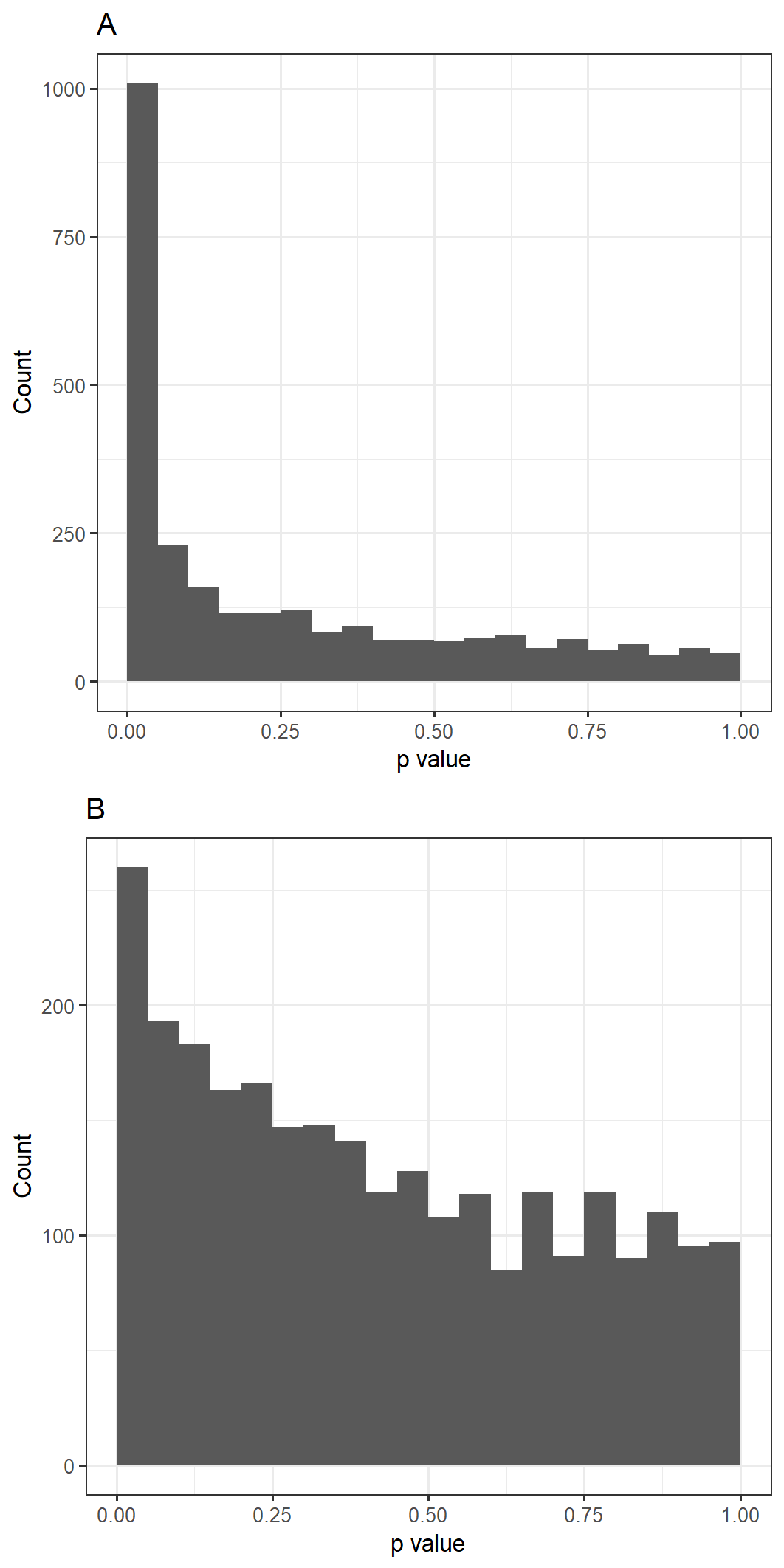


Supplementary Figure S2. Histogram of p-values for a significant difference between control and GLY treated protein abundance in MDR9 (A) and in B1045 (B). The distribution of p values shows that the MDR9 isolates include a large number of proteins with unambiguous change in abundance. This is demonstrated by the first bar in that histogram being more than 4 times the size of the next largest bar. The distribution for the B1045 isolates shows that many fewer proteins have an unambiguous change in abundance but that in the set as a whole, several hundred proteins do have modest change in abundance.
